# Supplementary material for: Gene Expression Profile of Uterine Leiomyoma from Women Exposed to Different Air Pollution Levels in Metropolitan Cities of Sao Paulo, Brazil
Source: Int J Mol Sci. 2023 Jan 26;24(3):2431. doi: 10.3390/ijms24032431 (PMC9917088; doi:10.3390/ijms24032431)
Supplement: Supplementary file 1 [file ijms-24-02431-s001.zip › ijms-2118746-supplementary.pdf]

Supplementary Materials

Gene Expression Profile of Uterine Leiomyoma from Women Exposed to Different Air Pollution Levels in Metropolitan Cities of Sao Paulo, Brazil

Laura Gonzalez dos Anjos,[1] Bruna Cristine de Almeida,[1] Edmund Chada Baracat,[1] Ayman Al-Hendy,[3] Qiwei Yang,[3] Katia Candido Carvalho,[1*]

[1] Laboratório de Ginecologia Estrutural e Molecular (LIM 58), Disciplina de Ginecologia, Departamento de Obstetricia e Ginecologia, Hospital das Clinicas da Faculdade de Medicina da Universidade de Sao Paulo (HCFMUSP), São Paulo 05403-010, Brazil
[2] Department of Obstetrics and Gynecology, University of Chicago, Chicago, IL 60637, USA
* Correspondence: carvalhokc@gmail.com; Tel.: +55-011-3061-7486

Table S1. *BCL2* gene expression association with patients ʻclinical features (Open array platform).

Variable	G1		G2		Total n (%)	p*
	Down n (%)	Up n (%)	Down n (%)	Up n (%)		
**Age (years)**						
≤40	1 (12.5)	2 (25)	3 (37.5)	2 (25)	8 (100)	0.5222
>40	5 (31)	2 (12.5)	3 (19)	6 (37.5)	16 (100)	
**Menarche (years)**						
≤12	2 (28.5)	1 (14.5)	2 (28.5)	2 (28.5)	7 (100)	0.9387
>12	4 (25)	3 (19)	3 (19)	6 (37)	16 (100)	
**Smoking**						
No	3 (19)	3 (19)	5 (31)	5 (31)	16 (100)	0.6397
Yes	3 (37.5)	1 (12.5)	1 (12.5)	3 (37.5)	8 (100)	
**Ethnicity**						
Caucasian	6 (33)	3 (17)	3 (17)	6 (33)	18 (100)	0.2615
Afrodescendant	0	1 (17)	3 (50)	2 (33)	6 (100)	
**Weight (Kg)**						
≤60	2 (28.5)	1 (14.5)	2 (28.5)	2 (28.5)	7 (100)	0.9773
>60	4 (23.5)	3 (18)	4 (23.5)	6 (35)	17 (100)	
**Body Mass Index**						
≤24.9	2 (20)	1 (10)	3 (30)	4 (40)	10 (100)	0.7943
>24.9	4 (29)	3 (21)	3 (21)	4 (29)	14 (100)	
**Uterine volume**						
≤ 646cm	2 (14)	2 (14)	4 (29)	6 (43)	14 (100)	0.4657
≥ 646cm	3 (37.5)	2 (25)	1 (12.5)	2 (25)	8 (100)	
**Pregnancy**						
0	1 (11)	2 (22)	4 (45)	2 (22)	9 (100)	0.2520
≥1	5 (33)	2 (13.5)	2 (13.5)	6 (40)	15 (100)	
**Abortion**						
No	4 (19)	5 (23)	6 (29)	6 (29)	21 (100)	0.3961
Yes	1 (33)	0	0	2 (67)	3 (100)	
**Contraceptive use**						
No	1 (12.5)	0	5 (62.5)	2 (25)	8 (100)	***0.0151***
Yes	5 (31)	4 (25)	1 (6)	6 (38)	16 (100)	
**LM treatment**						
No	5 (36)	3 (21)	4 (29)	2 (14)	14 (100)	0.1203
Yes	1 (10)	1 (10)	2 (20)	6 (60)	10 (100)	

**Surgery**						
Myomectomy	0	1 (33.3)	1 (33.3)	1 (33.3)	3 (100)	0.6768
Hysterectomy	6 (29)	3 (14)	5 (24)	7 (33)	21 (100)	
**Associated pathology**						
No	5 (28)	2 (11)	5 (28)	6 (33)	18 (100)	0.6198
Yes	1 (17)	2 (33)	1 (17)	2 (33)	6 (100)	

*Chi-square test.

Table S2. *BMP4* gene expression association with patients 'clinical features (Open array platform).

Variable	G1		Group 2		Total n (%)	p*
	Down n (%)	Up n (%)	Down n (%)	Up n (%)		
**Age (years)**						
≤40	1 (11)	3 (33.5)	3 (33.5)	2 (22)	9 (100)	0.2554
>40	5 (36)	1 (7)	3 (21)	5 (36)	14 (100)	
**Menarche (years)**						
≤12	3 (43)	0	2 (28.5)	2 (28.5)	7 (100)	0.3964
>12	3 (20)	4 (27)	3 (20)	5 (33)	15 (100)	
**Smoking**						
No	2 (13)	4 (27)	4 (27)	5 (33)	15 (100)	0.1757
Yes	4 (50)	0	2 (25)	2 (25)	8 (100)	
**Ethnicity**						
Caucasian	5 (29)	4 (24)	2 (12)	6 (35)	17 (100)	0.0624
Afrodescendant	1 (17)	0	4 (66)	1 (17)	6 (100)	
**Weight (Kg)**						
≤60	1 (14.5)	2 (28.5)	2 (28.5)	2 (28.5)	7 (100)	0.7302
>60	5 (31)	2 (13)	4 (25)	5 (31)	16 (100)	
**Body Mass Index**						
≤24.9	1 (10)	2 (20)	4 (40)	3 (30)	10 (100)	0.3708
>24.9	5 (39)	2 (15)	2 (15)	4 (31)	13 (100)	
**Uterine volume**						
≤ 646cm	3 (20)	2 (13)	5 (33.5)	5 (33.5)	15 (100)	0.6979
≥ 646cm	2 (28.5)	2 (28.5)	1 (14.5)	2 (28.5)	7 (100)	
**Pregnancy**						
0	0	3 (33.3)	3 (33.3)	3 (33.3)	9 (100)	0.0955
≥1	6 (43)	1 (7)	3 (21)	4 (29)	14 (100)	
**Abortion**						
No	6 (30)	3 (15)	5 (25)	6 (30)	20 (100)	***0.0342***
Yes	0	1 (33.3)	1 (33.3)	1 (33.3)	3 (100)	
**Contraceptive use**						
No	5 (42)	1 (8)	3 (25)	3 (25)	12 (100)	0.2870
Yes	1 (9)	3 (27.3)	3 (27.3)	4 (36.4)	11 (100)	
**LM treatment**						
No	5 (39)	3 (23)	2 (15)	3 (23)	13 (100)	0.2451
Yes	1 (10)	1 (10)	4 (40)	4 (40)	10 (100)	
**Surgery**						
Myomectomy	0	1 (25)	1 (25)	2 (50)	4 (100)	0.5651
Hysterectomy	6 (32)	3 (16)	5 (26)	5 (26)	19 (100)	
**Associated pathology**						
No	5 (29.4)	2 (11.8)	5 (29.4)	5 (29.4)	17 (100)	0.6235
Yes	1 (17)	2 (33)	1 (17)	2 (33)	6 (100)	

*Chi-square test.

Table S3. *DVL1* gene expression association with patients 'clinical features (Open array platform).

Variable	G1		G2		Total n (%)	p*
	Down n (%)	Up n (%)	Down n (%)	Up n (%)		
**Age (years)**						
≤40	1 (11.1)	3 (33.3)	2 (22.2)	3 (33.3)	9 (100)	0.3136
>40	5 (33)	1 (7)	4 (27)	5 (33)	15 (100)	
**Menarche (years)**						
≤12	2 (28.6)	1 (14.2)	2 (28.6)	2 (28.6)	7 (100)	0.9773
>12	4 (23.5)	3 (18)	4 (23.5)	6 (35)	17 (100)	
**Smoking**						
No	3 (19)	3 (19)	4 (25)	6 (37)	16 (100)	0.7710
Yes	3 (37.5)	1 (12.5)	2 (25)	2 (25)	8 (100)	
**Ethnicity**						
Caucasian	6 (33)	3 (17)	3 (17)	6 (33)	18 (100)	0.2615
Afrodescendant	0	1 (17)	3 (50)	2 (33)	6 (100)	
**Weight (Kg)**						
≤60	1 (14)	2 (29)	3 (43)	1 (14)	7 (100)	0.3043
>60	5 (29)	2 (12)	3 (18)	7 (41)	17 (100)	
**Body Mass Index**						
≤24.9	1 (10)	2 (20)	4 (40)	3 (30)	10 (100)	0.3537
>24.9	5 (36)	2 (14)	2 (14)	5 (36)	14 (100)	
**Uterine volume**						
≤ 646cm	1 (14)	0	3 (43)	3 (43)	7 (100)	0.2631
≥ 646cm	5 (33)	3 (20)	2 (14)	5 (33)	15 (100)	
**Pregnancy**						
0	1 (11.1)	2 (22.2)	4 (44.5)	2 (22.2)	9 (100)	0.2520
≥1	5 (33.5)	2 (13.3)	2 (13.3)	6 (40)	15 (100)	
**Abortion**						
No	5 (24)	4 (19)	6 (28.5)	6 (28.5)	21 (100)	0.4459
Yes	1 (33)	0	0	2 (67)	3 (100)	
**Contraceptive use**						
No	1 (12.5)	0	5 (62.5)	2 (25)	8 (100)	***0.0208***
Yes	5 (31)	4 (25)	1 (6)	6 (38)	16 (100)	
**LM treatment**						
No	5 (36)	3 (21)	4 (29)	2 (14)	14 (100)	0.1203
Yes	1 (10)	1 (10)	2 (20)	6 (60)	10 (100)	
**Surgery**						
Myomectomy	1 (17)	1 (17)	2 (33)	2 (33)	6 (100)	0.9309
hysterectomy	5 (28)	3 (17)	4 (22)	6 (33)	18 (100)	
**Associated pathology**						
No	4 (22)	3 (17)	4 (22)	7 (39)	18 (100)	0.7744
Yes	2 (33)	1 (17)	2 (33)	1 (17)	6 (100)	

*Chi-square test.

Table S4. *ESR1* gene expression association with patients 'clinical features (Open array platform).

Variable	G1		G2		Total n (%)	p*
	Down	Up	Down	Up		

	n (%)	n (%)	n (%)	n (%)		
**Age (years)**						
≤40	3 (30)	1 (10)	3 (30)	3 (30)	10 (100)	0.9723
>40	4 (31)	2 (15)	3 (23)	4 (31)	13 (100)	
**Menarche (years)**						
≤12	2 (29)	1 (14)	3 (43)	1 (14)	7 (100)	0.4138
>12	5 (34)	2 (13)	2 (13)	6 (40)	15 (100)	
**Smoking**						
No	5 (33)	1 (7)	4 (27)	5 (33)	15 (100)	0.6621
Yes	2 (25)	2 (25)	2 (25)	2 (25)	8 (100)	
**Ethnicity**						
Caucasian	7 (41)	2 (12)	2 (12)	6 (35)	17 (100)	***0.0424***
Afrodescendant	0	1 (17)	4 (66)	1 (17)	6 (100)	
**Weight (Kg)**						
≤60	2 (28.5)	1 (14.5)	2 (28.5)	2 (28.5)	7 (100)	0.9963
>60	5 (31)	2 (13)	4 (25)	5 (31)	16 (100)	
**Body Mass Index**						
≤24.9	2 (20)	1 (10)	4 (40)	3 (30)	10 (100)	0.5575
>24.9	5 (39)	2 (15)	2 (15)	4 (31)	13 (100)	
**Uterine volume**						
≤ 646cm	3 (21)	1 (7)	5 (36)	5 (36)	14 (100)	***0.0205***
≥ 646cm	4 (50)	1 (12.5)	1 (12.5)	2 (25)	8 (100)	
**Pregnancy**						
0	3 (33.3)	0	3 (33.3)	3 (33.3)	9 (100)	0.5110
≥1	4 (29)	3 (21)	3 (21)	4 (29)	14 (100)	
**Abortion**						
No	6 (35)	0	5 (30)	6 (35)	17 (100)	0.6862
Yes	1 (16.7)	3 (50)	1 (16.7)	1 (16.7)	6 (100)	
**Contraceptive use**						
No	1 (14)	0	3 (43)	3 (43)	7 (100)	0.2874
Yes	6 (37)	3 (19)	3 (19)	4 (25)	16 (100)	
**LM treatment**						
No	6 (46)	2 (15.5)	2 (15.5)	3 (23)	13 (100)	0.2216
Yes	1 (10)	1 (10)	4 (40)	4 (40)	10 (100)	
**Surgery**						
Myomectomy	2 (33.33)	0	2 (33.33)	2 (33.33)	6 (100)	0.7370
Hysterectomy	5 (29)	3 (18)	4 (24)	5 (29)	17 (100)	
**Associated pathology**						
No	5 (29.4)	2 (11.8)	5 (29.4)	5 (29.4)	17 (100)	0.9397
Yes	2 (33)	1 (17)	1 (17)	2 (33)	6 (100)	

*Chi-square test.

Table S5. *FGFR3* gene expression association with patients ʻclinical features (Open array platform).

**Variable**	**G1**		**G2**		**Total n (%)**	**p***
	**Down n (%)**	**Up n (%)**	**Down n (%)**	**Up n (%)**		
**Age (years)**						
≤40	1 (11.1)	3 (33.3)	4 (44.4)	1 (11.1)	9 (100)	0.3370
>40	4 (27)	2 (13)	4 (27)	5 (33)	15 (100)	
**Menarche (years)**						
≤12	3 (43)	0	3 (43)	1 (14)	7 (100)	0.1659
>12	2 (12)	5 (29)	5 (29)	5 (29)	17 (100)	

**Smoking**						
No	3 (19)	3 (19)	5 (31)	5 (31)	16 (100)	0.7982
Yes	2 (25)	2 (25)	3 (37.5)	1 (12.5)	8 (100)	
**Ethnicity**						
Caucasian	4 (22)	5 (28)	4 (22)	5 (28)	18 (100)	0.2016
Afrodescendant	1 (17)	0	4 (66)	1 (17)	6 (100)	
**Weight (Kg)**						
≤60	0	3 (43)	2 (28.5)	2 (28.5)	7 (100)	0.2143
>60	5 (29)	2 (12)	6 (35)	4 (24)	17 (100)	
**Body Mass Index**						
≤24.9	0	3 (30)	4 (40)	3 (30)	10 (100)	0.1982
>24.9	5 (36)	2 (14)	4 (29)	3 (21)	14 (100)	
**Uterine volume**						
≤ 646cm	3 (21)	1 (7)	5 (36)	5 (36)	14 (100)	0.2841
≥ 646cm	2 (25)	3 (37.5)	2 (25)	1 (12.5)	8 (100)	
**Pregnancy**						
0	0	3 (33.3)	4 (44.4)	2 (22.2)	9 (100)	0.1986
≥1	5 (33)	2 (13)	4 (27)	4 (27)	15 (100)	
**Abortion**						
No	4 (19)	5 (24)	8 (38)	4 (19)	21 (100)	0.2127
Yes	1 (33)	0	0	2 (67)	3 (100)	
**Contraceptive use**						
No	1 (12.5)	0	6 (75)	1 (12.5)	8 (100)	***0.0194***
Yes	4 (25)	5 (31)	2 (13)	5 (31)	16 (100)	
**LM treatment**						
No	4 (28.5)	4 (28.5)	5 (36)	1 (7)	14 (100)	0.0990
Yes	1 (10)	1 (10)	3 (30)	5 (50)	10 (100)	
**Surgery**						
Myomectomy	0	2 (33)	3 (50)	1 (17)	6 (100)	0.3683
Hysterectomy	5 (28)	3 (16)	5 (28)	5 (28)	18 (100)	
**Associated pathology**						
No	3 (17)	4 (22)	7 (39)	4 (22)	18 (100)	0.6695
Yes	2 (33)	1 (17)	1 (17)	2 (33)	6 (100)	

*Chi-square test.

Table S6. *GREM1* gene expression association with patients ‘clinical features (Open array platform).

Variable	G1		G2		Total n (%)	p*
	Down n (%)	Up n (%)	Down n (%)	Up n (%)		
**Age (years)**						
≤40	3 (33.3)	1 (11.1)	4 (44.4)	1 (11.1)	9 (100)	0.3370
>40	2 (13)	4 (27)	4 (27)	5 (33)	15 (100)	
**Menarche (years)**						
≤12	3 (43)	0	2 (28.5)	2 (28.5)	7 (100)	0.2053
>12	2 (12.5)	5 (31)	6 (37.5)	3 (19)	16 (100)	
**Smoking**						
No	4 (25)	2 (12.5)	6 (37.5)	4 (25)	16 (100)	0.5222
Yes	1 (12.5)	3 (37.5)	2 (25)	2 (25)	8 (100)	
**Ethnicity**						
Caucasian	4 (22)	5 (28)	5 (28)	4 (22)	18 (100)	0.4536
Afrodescendant	1 (17)	0	3 (50)	2 (33)	6 (100)	

**Weight (Kg)**						
≤60	2 (28.5)	1 (14.5)	2 (28.5)	2 (28.5)	7 (100)	0.8953
>60	3 (18)	4 (23.5)	6 (35)	4 (23.5)	17 (100)	
**Body Mass Index**						
≤24.9	2 (20)	1 (10)	4 (40)	3 (30)	10 (100)	0.7122
>24.9	3 (21)	4 (29)	4 (29)	3 (21)	14 (100)	
**Uterine volume**						
≤ 646cm	3 (21)	1 (7)	6 (43)	4 (29)	14 (100)	0.2501
≥ 646cm	2 (25)	3 (37.5)	1 (12.5)	2 (25)	8 (100)	
**Pregnancy**						
0	2 (22.2)	1 (11.1)	3 (33.3)	3 (33.3)	9 (100)	0.7851
≥1	3 (20)	4 (27)	5 (33)	3 (20)	15 (100)	
**Abortion**						
No	5 (24)	4 (19)	7 (33)	5 (24)	21 (100)	0.7851
Yes	0	1 (33.3)	1 (33.3)	1 (33.3)	3 (100)	
**Contraceptive use**						
No	0	1 (12.5)	4 (50)	3 (37.5)	8 (100)	0.1993
Yes	5 (31)	4 (25)	4 (25)	3 (19)	16 (100)	
**LM treatment**						
No	4 (28.5)	4 (28.5)	4 (28.5)	2 (14.5)	14 (100)	0.2954
Yes	1 (10)	1 (10)	4 (40)	4 (40)	10 (100)	
**Surgery**						
Myomectomy	1 (16.67)	1 (16.67)	3 (50)	1 (16.67)	6 (100)	0.7959
Hysterectomy	4 (22)	4 (22)	5 (28)	5 (28)	18 (100)	
**Associated pathology**						
No	5 (28)	2 (11)	8 (44)	3 (17)	18 (100)	***0.0223***
Yes	0	3 (50)	0	3 (50)	6 (100)	

*Chi-square test.

Table S7. *HHAT* gene expression association with patients 'clinical features (Open array platform).

Variable	Group 1		Group 2		Total n (%)	p*
	**Down n (%)**	**Up n (%)**	**Down n (%)**	**Up n (%)**		
**Age (years)**						
≤40	2 (25)	1 (12.5)	3 (37.5)	2 (25)	8 (100)	0.4575
>40	6 (37.5)	1 (6)	2 (12.5)	7 (44)	16 (100)	
**Menarche (years)**						
≤12	3 (43)	0	2 (28.5)	2 (28.5)	7 (100)	0.5573
>12	5 (31)	2 (12.5)	2 (12.5)	7 (44)	16 (100)	
**Smoking**						
No	5 (31)	1 (6)	4 (25)	6 (38)	16 (100)	0.8703
Yes	3 (37.5)	1 (12.5)	1 (12.5)	3 (37.5)	8 (100)	
**Ethnicity**						
Caucasian	7 (39)	2 (11)	1 (6)	8 (44)	18 (100)	***0.0160***
Afrodescendant	1 (17)	0	4 (66)	1 (17)	6(100)	
**Weight (Kg)**						
≤60	2 (28.5)	1 (14.5)	2 (28.5)	2 (28.5)	7 (100)	0.8057
>60	6 (35)	1 (6)	3 (18)	7 (41)	17 (100)	
**Body Mass Index**						
≤24.9	2 (20)	1 (10)	2 (20)	5 (50)	10 (100)	0.6388
>24.9	6 (43)	1 (7)	3 (21)	4 (29)	14 (100)	

**Uterine volume**						
≤ 646cm	4 (28.5)	0	4 (28.5)	6 (43)	14 (100)	0.1940
≥ 646cm	4 (50)	1 (12.5)	0	3 (37.5)	8 (100)	
**Pregnancy**						
0	2 (22.2)	1 (11.1)	3 (33.3)	3 (33.3)	9 (100)	0.6120
≥1	6 (40)	1 (7)	2 (13)	6 (40)	15 (100)	
**Abortion**						
No	7 (33)	2 (10)	5 (24)	7 (33)	21 (100)	0.6198
Yes	1 (33)	0	0	2 (67)	3 (100)	
**Contraceptive use**						
No	1 (12.5)	0	3 (37.5)	4 (50)	8 (100)	***0.0194***
Yes	7 (44)	2 (12.5)	2 (12.5)	5 (31)	16 (100)	
**LM treatment**						
No	6 (43)	2 (14)	2 (14)	4 (29)	14 (100)	0.2899
Yes	2 (20)	0	3 (30)	5 (50)	10 (100)	
**Surgery**						
Myomectomy	1 (17)	1 (17)	2 (33)	2 (33)	6 (100)	0.5786
Hysterectomy	7 (39)	1 (5)	3 (17)	7 (39)	18 (100)	
**Associated pathology**						
No	6 (33)	1 (6)	4 (22)	7 (39)	18 (100)	0.8565
Yes	2 (33)	1 (17)	1 (17)	2 (33)	6 (100)	

*Chi-square test.

Table S8. *WNT5B* gene expression association with patients ʻclinical features (Open array platform).

**Variable**	**G1**		**G2**		**Total n (%)**	**p***
	**Down n (%)**	**Up n (%)**	**Down n (%)**	**Up n (%)**		
**Age (years)**						
≤40	2 (22.2)	2 (22.2)	4 (44.4)	1 (11.1)	9 (100)	0.6434
>40	3 (20)	3 (20)	4 (27)	5 (33)	15 (100)	
**Menarche (years)**						
≤12	3 (43)	0	2 (28.5)	2 (28.5)	7 (100)	0.2321
>12*	2 (13)	5 (31)	5 (31)	4 (25)	16 (100)	
**Smoking**						
No	3 (21.4)	3 (21.4)	3 (21.4)	5 (35.8)	14 (100)	0.3943
Yes	2 (20)	2 (20)	5 (50)	1 (10)	10 (100)	
**Ethnicity**						
Caucasian	4 (22)	5 (28)	4 (22)	5 (28)	18 (100)	0.2016
Afrodescendant	1 (17)	0	4 (66)	1 (17)	6 (100)	
**Weight (Kg)**						
≤60	2 (29)	1 (14)	3 (43)	1 (14)	7 (100)	0.7506
>60	3 (18)	4 (24)	5 (29)	5 (29)	17 (100)	
**Body Mass Index**						
≤24.9	2 (20)	1 (10)	4 (40)	3 (30)	10 (100)	0.7122
>24.9	3 (21)	4 (29)	4 (29)	3 (21)	14 (100)	
**Uterine volume**						
≤ 646cm	3 (21)	1 (7)	5 (36)	5 (36)	14 (100)	0.1370
≥ 646cm	1 (12.5)	4 (50)	2 (25)	1 (12.5)	8 (100)	
**Pregnancy**						
0	1 (11.1)	2 (22.2)	5 (55.5)	1 (11.1)	9 (100)	0.2712
≥1	4 (27)	3 (20)	3 (20)	5 (33)	15 (100)	

**Abortion**						
No	5 (24)	4 (19)	8 (38)	4 (19)	21 (100)	0.2127
Yes	0	1 (33)	0	2 (67)	3 (100)	
**Contraceptive use**						
No	0	1 (12.5)	6 (75)	1 (12.5)	8 (100)	***0.0194***
Yes	5 (31)	4 (25)	2 (13)	5 (31)	16 (100)	
**LM treatment**						
No	5 (36)	3 (21)	5 (36)	1 (7)	14 (100)	***0.0477***
Yes	0	2 (20)	3 (30)	5 (50)	10 (100)	
**Surgery**						
Myomectomy	1 (16.67)	1 (16.67)	3 (50)	1 (16.67)	6 (100)	0.7959
Hysterectomy	4 (22)	4 (22)	5 (28)	5 (28)	18 (100)	
**Associated pathology**						
No	5 (28)	2 (11)	6 (33)	5 (28)	18 (100)	0.1608
Yes	0	3 (50)	2 (33)	1 (17)	6 (100)	

*Chi-square test.

Table S9. *LITAF* gene expression association with patients ´clinical features (Array Qiagen).

Variable	G1		G2		Total n (%)	p*
	Down n (%)	Up n (%)	Down n (%)	Up n (%)		
**Age (years)**						
≤40	1 (33)	0	2 (67)	0	3 (100)	0.2615
>40	1 (11.1)	2 (22.2)	2 (22.2)	4 (44.4)	9 (100)	
**Menarche (years)**						
≤12	1 (25)	0	2 (50)	1 (25)	4 (100)	0.4373
>12	1 (14)	2 (29)	1 (14)	3 (43)	7 (100)	
**Smoking**						
No	2 (29)	0	3 (42)	2 (29)	7 (100)	0.1870
Yes	0	2 (40)	1 (20)	2 (40)	5 (100)	
**Ethnicity**						
Caucasian	1 (12.5)	2 (25)	1 (12.5)	4 (50)	8 (100)	0.0947
Afrodescendant	1 (25)	0	3 (75)	0	4 (100)	
**Weight (Kg)**						
≤70	0	1 (25)	0	3 (75)	4 (100)	0.0947
>70	2 (25)	1 (12.5)	4 (50)	1 (12.5)	8 (100)	
**Body Mass Index**						
≤24.9	0	0	0	3 (100)	3 (100)	***0.0460***
>24.9	2 (22.2)	2 (22.2)	4 (44.4)	1 (11.1)	9 (100)	
**Uterine volume**						
≤ 646cm	0	1 (14)	4 (57)	2 (29)	7 (100)	0.1203
≥ 646cm	2 (40)	1 (20)	0	2 (40)	5 (100)	
**Pregnancy**						
0	0	0	2 (100)	0	2 (100)	0.1870
≥1	2 (20)	2 (20)	2 (20)	4 (40)	10 (100)	
**Abortion**						
No	1 (11.1)	2 (22.2)	3 (33.3)	3 (33.3)	9 (100)	0.7212
Yes	1 (33.3)	0	1 (33.3)	1 (33.3)	3 (100)	
**Contraceptive use**						
No	1 (20)	0	2 (40)	2 (40)	5 (100)	0.6338
Yes	1 (14.5)	2 (28.5)	2 (28.5)	2 (28.5)	7 (100)	

**LM treatment**						
No	2 (33)	1 (17)	1 (17)	2 (33)	6 (100)	0.3916
Yes	0	1 (17)	3 (50)	2 (33)	6 (100)	
**Surgery**						
Myomectomy	0	0	1 (100)	0	1 (100)	0.5355
Hysterectomy	2 (18.2)	2 (18.2)	3 (27.2)	4 (36.3)	11 (100)	
**Associated pathology**						
No	2 (22.2)	1 (11.1)	3 (33.3)	3 (33.3)	9 (100)	0.7212
Yes	0	1 (33.3)	1 (33.3)	1 (33.3)	3 (100)	

*Chi-square test.

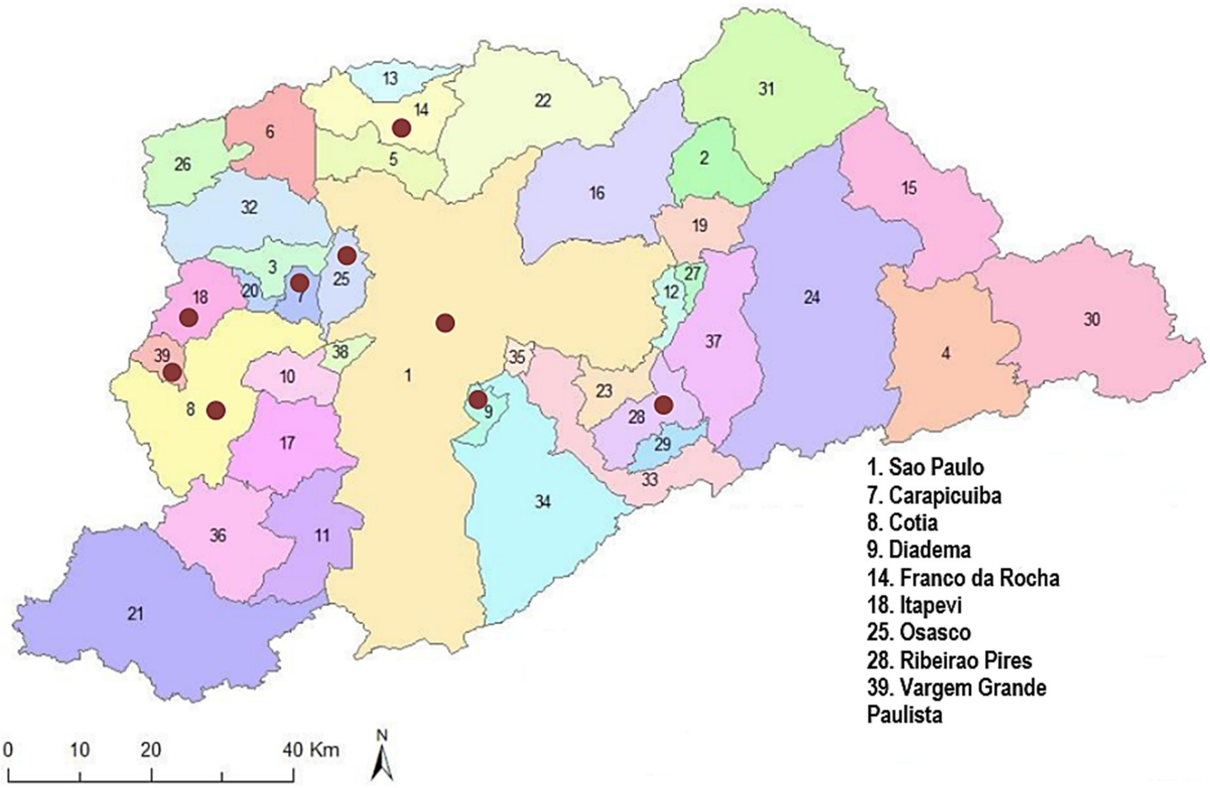

Figure S1. Representative Map from the metropolitan region of Sao Paulo. The red dots indicate the cities from the selected patients. The number and name of the Cities are indicated too.

Table S10. Genes included in the array plate analysis of the gene's expression profile (SYBR green® platform CLAH27251A).

Gene symbol	Gene name	RefSeq
MXRA5	Matrix-remodelling associated 5	NM_015419
IL1RN	Interleukin 1 receptor antagonist	NM_000577
TIMP3	TIMP metallopeptidase inhibitor 3	NM_000362
MMP2	Matrix metallopeptidase 2	NM_004530
MMP9	Matrix metallopeptidase 9	NM_004994
COMP	Cartilage oligomeric matrix protein	NM_000095
MMP26	Matrix metallopeptidase 26	NM_021801
POSTN	Periostin, osteoblast specific factor	NM_006475
OLFM4	Olfactomedin 4	NM_006418
SLC2A6	Solute carrier family 2 (facilitated glucose transporter), member 6	NM_017585
IGFBP5	Insulin-like growth factor binding protein 5	NM_000599
RARB	Retinoic acid receptor, beta	NM_000965
IGFBP1	Insulin-like growth factor binding protein 1	NM_000596
COL6A2	Collagen, type VI, alpha 2	NM_001849
CTHRC1	Collagen triple helix repeat containing 1	NM_138455
COL1A1	Collagen, type I, alpha 1	NM_000088
COL6A3	Collagen, type VI, alpha 3	NM_004369
SFRP4	Secreted frizzled-related protein 4	NM_003014
MCL1	Myeloid cell leukemia sequence 1 (BCL2-related)	NM_021960
CASP8	Caspase 8, apoptosis-related cysteine peptidase	NM_001228
WNT5A	Wingless-type MMTV integration site family, member 5A	NM_003392

MUC16	Mucin 16, cell surface associated	NM_024690
THBS2	Thrombospondin 2	NM_003247
DPP4	Dipeptidyl-peptidase 4	NM_001935
DUOX1	Dual oxidase 1	NM_175940
PDGFRB	Platelet-derived growth factor receptor, beta polypeptide	NM_002609
PLXNA3	Plexin A3	NM_017514
EFNA1	Ephrin-A1	NM_182685
LRP4	Low density lipoprotein receptor-related protein 4	NM_002334
TGFBR3	Transforming growth factor, beta receptor III	NM_003243
CTNNA2	Catenin (cadherin-associated protein), alpha 2	NM_004389
HABP2	Hyaluronan binding protein 2	NM_004132
IFNGR2	Interferon gamma receptor 2 (interferon gamma transducer 1)	NM_005534
PCNA	Proliferating cell nuclear antigen	NM_182649
ERRFI1	ERBB receptor feedback inhibitor 1	NM_018948
LITAF	Lipopolysaccharide-induced TNF factor	NM_004862
CXCL14	Chemokine (C-X-C motif) ligand 14	NM_004887
CFD	Complement factor D (adipsin)	NM_001928
KLRC1	Killer cell lectin-like receptor subfamily C, member 1	NM_002259
PER2	Period homolog 2 (Drosophila)	NM_022817
CDK6	Cyclin-dependent kinase 6	NM_001259
ERBB2	V-erb-b2 erythroblastic leukemia viral oncogene homolog 2	NM_004448
MTNR1A	Melatonin receptor 1A	NM_005958
CCNG1	Cyclin G1	NM_004060
MTNR1B	Melatonin receptor 1B	NM_005959
ACTBL2	Actin, beta-like 2	NM_001017992
UBE2A	Ubiquitin-conjugating enzyme E2A	NM_003336
GADD45A	Growth arrest and DNA-damage-inducible, alpha	NM_001924
FOXC2	Forkhead box C2 (MFH-1, mesenchyme forkhead 1)	NM_005251
CENPE	Centromere protein E, 312kDa	NM_001813
CSRP2	Cysteine and glycine-rich protein 2	NM_001321
KISS1R	KISS1 receptor	NM_032551
ESR1	Estrogen receptor 1	NM_000125
ESR2	Estrogen receptor 2 (ER beta)	NM_001437
PRLR	Prolactin receptor	NM_000949
TSHR	Thyroid stimulating hormone receptor	NM_000369
GAST	Gastrin	NM_000805
PAEP	Progestagen-associated endometrial protein	NM_002571
CLOCK	Clock homolog (mouse)	NM_004898
NR4A2	Nuclear receptor subfamily 4, group A, member 2	NM_006186
LAMB3	Laminin, beta 3	NM_000228
SLC1A1	Solute carrier family 1 member 1	NM_004170
SLC15A1	Solute carrier family 15 (oligopeptide transporter), member 1	NM_005073
TCN1	Transcobalamin I (vitamin B12 binding protein, R binder family)	NM_001062
SLC16A6	Solute carrier family 16, member 6 (monocarboxylic acid transporter 7)	NM_004694
PTGER2	Prostaglandin E receptor 2 (subtype EP2), 53kDa	NM_000956
HPGD	Hydroxyprostaglandin dehydrogenase 15-(NAD)	NM_000860
GAB1	GRB2-associated binding protein 1	NM_002039
SORD	Sorbitol dehydrogenase	NM_003104
DHRS3	Dehydrogenase/reductase (SDR family) member 3	NM_004753
C4BPA	Complement component 4 binding protein, alpha	NM_000715
CLU	Clusterin	NM_001831

PENK	Proenkephalin	NM_006211
HLA-DOB	Major histocompatibility complex, class II, DO beta	NM_002120
S100P	S100 calcium binding protein P	NM_005980
CALB2	Calbindin 2	NM_001740
CYP3A5	Cytochrome P450, family 3, subfamily A, polypeptide 5	NM_000777
SERPING1	Serpin peptidase inhibitor, clade G (C1 inhibitor), member 1	NM_000062
CRISP3	Cysteine-rich secretory protein 3	NM_006061
LIF	Leukemia inhibitory factor (cholinergic differentiation factor)	NM_002309
GPX3	Glutathione peroxidase 3 (plasma)	NM_002084
HOXA10	Homeobox A10	NM_018951
HOXA11	Homeobox A11	NM_005523
GAPDH*	Glyceraldehyde-3-phosphate dehydrogenase	NM_002046
TFRC*	Transferrin receptor (p90, CD71)	NM_003234
HPRT1*	Hypoxanthine phosphoribosyltransferase 1	NM_000194
LDHA*	Lactate dehydrogenase A	NM_005566
ACTB*	Actin, beta	NM_001101

*Housekeeping genes included as endogenous control of the gene expression levels.

Table S11. List of genes plotted as differentially expressed in G1 and G2 versus reference group in the scatter plots*.

G1 x Reference Group				G2 x Reference Group			
Upregulated		Downregulated		Upregulated		Downregulated	
**Gene**	**Fold C**	Gene	**Fold C**	**Gene**	**Fold C**	***Gene***	**Fold C**
EIF5A	3.34	*IL1RN*	-11.29	*EIF5A*	4.06	*IL1RN*	-16.07
IGFBP5	2.57	*MMP9*	-3.75	*RARB*	3.87	*MMP9*	-3.19
RARB	4.65	*COMP*	-6.96	*COL6A3*	2.07	*COMP*	-10.91
COL6A3	2.44	*MMP26*	-95.80	*SFRP4*	3.50	*MMP26*	-163.02
ERRFI1	3.82	*OLFM4*	-30.13	*PLXNA3*	2.12	*OLFM4*	-60.42
LITAF	3.68	*SLC2A6*	-13.26	*TGFBR3*	2.75	*SLC2A6*	-16.38
CXCL14	6.91	*IGFBP1*	-95.80	*ERRFI1*	2.88	*IGFBP1*	-127.74
UBE2A	2.41	*MCL1*	-2.63	*LITAF*	5.86	*WNT5A*	-2.69
CSRP2	4.02	*MUC16*	-34.63	*CXCL14*	2.00	*MUC16*	-39.95
ESR1	2.77	*DPP4*	-5.19	*UBE2A*	2.71	*DPP4*	-3.41
PTGER2	3.04	*CTNNA2*	-7.00	*CSRP2*	5.64	*DUOX1*	-4.20
SORD	3.67	*HABP2*	-67.16	*ESR1*	3.05	*PDGFRB*	-2.07
HOXA10	12.49	*CFD*	-13.80	*PTGER2*	2.94	*CTNNA2*	-14.55
HOXA11	2.24	*KLRC1*	-52.23	*SORD*	4.15	*HABP2*	-229.36
LDHA	3.14	*ERBB2*	-2.66	*HOXA10*	10.01	*CFD*	-11.32
		MTNR1A	-62.09	*HOXA11*	2.76	*KLRC1*	-7.76
		MTNR1B	-142.19	*LDHA*	2.77	*ERBB2*	-2.17
		ACTBL2	-95.80			*MTNR1A*	-229.36
		GADD45A	-6.30			*MTNR1B*	-131.67
		FOXC2	-50.24			*ACTBL2*	-225.84
		CENPE	-5.71			*GADD45A*	-8.93
		KISS1R	-95.80			*FOXC2*	-43.31
		ESR2	-14.15			*CENPE*	-4.83
		TSHR	-46.53			*KISS1R*	-229.36
		GAST	-95.80			*ESR2*	-4.73
		PAEP	-95.80			*TSHR*	-16.35
		NR4A2	-7.13			*GAST*	-141.86
		LAMB3	-78.80			*PAEP*	-89.22
		SLC15A1	-22.73			*NR4A2*	-4.60
		TCN1	-14.99			*LAMB3*	-75.34
		SLC16A6	-3.28			*SLC15A1*	-18.59

DHRS3	-5.04	*TCN1*	-17.75
C4BPA	-40.70	*DHRS3*	-6.90
HLA-DOB	-21.85	*C4BPA*	-17.17
S100P	-11.32	*PENK*	-20.58
CALB2	-13.43	*HLA-DOB*	-10.69
CYP3A5	-2.63	*S100P*	-16.66
CRISP3	-84.28	*CALB2*	-37.23
LIF	-22.41	*CYP3A5*	-4.27
		CRISP3	-45.75
		LIF	-48.08

*Figures 5a and b on the manuscript

Table S12. Genes included in the Open array analysis of the gene expression profile (TaqMan® platform).

Gene symbol	Gene name	*TaqMan®* assay	RefSeq
ACTB*	Actin, beta	hs 99999903_m1	NM_001101
B2M*	Beta-2-microglobulin	hs 00984230_m1	NM_004048
GAPDH*	Glyceraldehyde-3-phosphate dehydrogenase	hs 99999905_m1	NM_002046
GUSB*	Glucuronidase, beta	hs 00939627_m1	NM_000181
HPRT1*	Hypoxanthine phosphoribosyltransferase 1	hs 02800695_m1	NM_000194
RPLP0*	Ribosomal protein, large, P0	hs 99999902_m1	NM_001002
APC	Adenomatous polyposis coli	hs 01568269_m1	NM_000038
AXIN1	Axin 1	hs 00394718_m1	NM_003502
AXIN2	Axin 2	hs 00610344_m1	NM_004655
BCL2	B-cell CLL/lymphoma 2	hs 00608023_m1	NM_000633
BMP2	Bone morphogenetic protein 2	hs 00154192_m1	NM_001200
BMP4	Bone morphogenetic protein 4	hs 01041266_m1	NM_130851
BMP5	Bone morphogenetic protein 5	hs 00234930_m1	NM_021073
BMP6	Bone morphogenetic protein 6	hs 01099594_m1	NM_001718
BMP7	Bone morphogenetic protein 7	hs 00233476_m1	NM_001719
BMP8B	Bone morphogenetic protein 8b	hs 01629120_s1	NM_001720
CCND1	Cyclin D1	hs 00765553_m1	NM_053056
CCND2	Cyclin D2	hs 00153380_m1	NM_001759
CSNK1A1	Casein kinase 1, alpha 1	hs 00793391_m1	NM_001892
CSNK2A1	Casein kinase 2, alpha 1 polypeptide	hs 00953536_m1	NM_001895
CTNNB1	Catenin (cadherin-associated protein), beta 1, 88kDa	hs 00355049_m1	NM_001904
CTNNBIP1	Catenin, beta interacting protein 1	hs 00172016_m1	NM_020248
DAAM1	Dishevelled associated activator of morphogenesis 1	hs 00323674_m1	NM_014992
DHH	Desert hedgehog	hs 00368306_m1	NM_021044
DISP1	Dispatched homolog 1 (Drosophila)	hs 00399179_m1	NM_032890
DISP2	Dispatched homolog 2 (Drosophila)	hs 00394338_m1	NM_033510
DVL1	Dishevelled, dsh homolog 1 (Drosophila)	hs 00182896_m1	NM_004421
DVL2	Dishevelled, dsh homolog 2 (Drosophila)	hs 00182901_m1	NM_004422
EIF5A	eucariotic initiation factor 5A	hs 04188519_m1	NM_001143
ERBB4	V-erb-a erythroblastic leukemia viral oncogene homolog 4 (avian)	hs 00955525_m1	NM_005235
ESR1	Estrogen receptor	hs 00174860_m1	NM_001253
FGF4	Fibroblast growth factor 4	hs 00999691_m1	NM_002007
FGF9	Fibroblast growth factor 9 (GLIa-activating factor)	hs 00181829_m1	NM_002010
FGFR3	Fibroblast growth factor receptor 3	hs 00179829_m1	NM_000142
FOXE1	Forkhead box E1 (thyroid transcription factor 2)	hs 00916085_s1	NM_004473

FOXN1	Forkhead box N1	hs 00186096_m1	NM_003593
FOXO3A	Forkhead box	hs 00818121_m1	NM_001455
FRZB	Frizzled-related protein	hs 00173503_m1	NM_001463
FZD1	Frizzled family receptor 1	hs 00268943_s1	NM_003505
FZD2	Frizzled family receptor 2	hs 00361432_s1	NM_001466
FZD4	Frizzled family receptor 4	hs 00201853_m1	NM_012193
FZD5	Frizzled family receptor 5	hs 00258278_s1	NM_003468
FZD6	Frizzled family receptor 6	hs 00171574_m1	NM_003506
FZD7	Frizzled family receptor 7	hs 00275833_s1	NM_003507
FZD8	Frizzled family receptor 8	hs 00259040_s1	NM_031866
FZD9	Frizzled family receptor 9	hs 00268952_s1	NM_003508
GAS1	Growth arrest-specific 1	hs 00266715_s1	NM_002048
GLI1	GLI family zinc finger 1	hs 01110766_m1	NM_005269
GLI2	GLI family zinc finger 2	hs 01119974_m1	NM_005270
GLI3	GLI family zinc finger 3	hs 00609233_m1	NM_000168
GREM1	Gremlin 1	hs 00171951_m1	NM_013372
GSK3A	Glycogen synthase kinase 3 alpha	hs 00997938_m1	NM_019884
GSK3B	Glycogen synthase kinase 3 beta	hs 01047719_m1	NM_002093
HHAT	Hedgehog acyltransferase	hs 00911326_m1	NM_018194
HHIP	Hedgehog interacting protein	hs 01011015_m1	NM_022475
IFT52	Intraflagellar transport 52 homolog (Chlamydomonas)	hs 00211198_m1	NM_016004
IHH	Indian hedgehog	hs 01081801_m1	NM_002181
JUN	Jun proto-oncogene	hs 01103582_s1	NM_002228
LATS1	LATS, large tumor suppressor, homolog 1 (Drosophila)	hs 01125528_m1	NM_004690
LATS2	LATS, large tumor suppressor, homolog 2 (Drosophila)	hs 00324396_m1	NM_014572
LEF1	Lymphoid enhancer-binding factor 1	hs 01547250_m1	NM_016269
MAPK1	Mitogen-activated protein kinase 1	hs 01046830_m1	NM_002745
MMP7	Matrix metallopeptidase 7 (matrilysin, uterine)	hs 01042796_m1	NM_002423
MAPK8	Mitogen-activated protein kinase 8	hs 00177083_m1	NM_002750
MTSS1	Metastasis suppressor 1	hs 00207341_m1	NM_014751
MYC	V-myc myelocytomatosis viral oncogene homolog (avian)	hs 00153408_m1	NM_002467
PGR	progesterone receptor	hs 01556702_m1	NM_000926
PRL	prolaction	hs 00168730_m1	NM_000948
PRLR	prolactin receptor	hs 01061477_m1	NM_000949
PTCH1	Patched 1	hs 00181117_m1	NM_000264
PTCH2	Patched 2	hs 00184804_m1	NM_003738
PTCHD1	Patched domain containing 1	hs 00288486_m1	NM_173495
PTCHD2	Patched domain containing 2	hs 01367724_m1	NM_020780
PTCHD3	Patched domain containing 3	hs 01584645_m1	NM_001034
PTEN	protein tensin homologue	hs 02621230_s1	NM_000314
RAB23	RAB23, member RAS oncogene family	hs 00212407_m1	NM_183227
RHOA	Ras homolog gene family, member A	hs 00357608_m1	NM_001664
SFRP1	Secreted frizzled-related protein 1	hs 00610060_m1	NM_003012
SFRP4	Secreted frizzled-related protein 4	hs 00180066_m1	NM_003014
SHH	Sonic hedgehog	hs 00179843_m1	NM_000193
SLC2A1	Facilitated glucose transporter member 1	hs 00892681_m1	NM_001455
SLC2A3	Facilitated glucose transporter member 3	hs 00359840_m1	NM_006931
SLC2A4	Facilitated glucose transporter member 4	hs 00168966_m1	NM_001042
SMO	Smoothened, frizzled family receptor	hs 01090242_m1	NM_005631
SUFU	Suppressor of fused homolog (Drosophila)	hs 00171981_m1	NM_016169

TCF7	Transcription factor 7 (T-cell specific, HMG-box)	hs 00175273_m1	NM_003202
TCF7L1	Transcription factor 7-like 1 (T-cell specific, HMG-box)	hs 01064103_m1	NM_031283
TLE1	Transducin-like enhancer of split 1 (E(sp1) homolog, Drosophila)	hs 00270768_m1	NM_005077
TP53	Tumor protein p53	hs 01034249_m1	NM_000546
VEGFA	Vascular endothelial growth factor A	hs 00900055_m1	NM_003376
WIF1	WNT inhibitory factor 1	hs 00183662_m1	NM_007191
WISP1	WNT1 inducible signaling pathway protein 1	hs 04234730_m1	NM_003882
WNT1	Wingless-type MMTV integration site family, member 1	hs 01011247_m1	NM_005430
WNT10A	Wingless-type MMTV integration site family, member 10A	hs 00228741_m1	NM_025216
WNT10B	Wingless-type MMTV integration site family, member 10B	hs 00559664_m1	NM_003394
WNT11	Wingless-type MMTV integration site family, member 11	hs 00182986_m1	NM_004626
WNT16	Wingless-type MMTV integration site family, member 16	hs 00365138_m1	NM_057168
WNT2	Wingless-type MMTV integration site family member 2	hs 00608224_m1	NM_003391
WNT2B	Wingless-type MMTV integration site family, member 2B	hs 00921614_m1	NM_004185
WNT3	Wingless-type MMTV integration site family, member 3	hs 00902257_m1	NM_030753
WNT3A	Wingless-type MMTV integration site family, member 3A	hs 00263977_m1	NM_033131
WNT4	Wingless-type MMTV integration site family, member 4	hs 01573504_m1	NM_030761
WNT5A	Wingless-type MMTV integration site family, member 5A	hs 00998537_m1	NM_003392
WNT5B	Wingless-type MMTV integration site family, member 5B	hs 01086864_m1	NM_032642
WNT6	Wingless-type MMTV integration site family, member	hs 00362452_m1	NM_006522
WNT7A	Wingless-type MMTV integration site family, member 7A	hs 01114990_m1	NM_004625
WNT7B	Wingless-type MMTV integration site family, member 7B	hs 00536497_m1	NM_058238
WNT8A	Wingless-type MMTV integration site family, member 8A	hs 00230534_m1	NM_058244
WNT8B	Wingless-type MMTV integration site family, member 8B	hs 00610126_m1	NM_003393
WNT9A	Wingless-type MMTV integration site family, member 9A	hs 00243321_m1	NM_003395
WNT9B	Wingless-type MMTV integration site family, member 9B	hs 00287409_m1	NM_003396

*Housekeeping genes included as endogenous control of the gene expression levels.
